# Progress in Neurology

**Published:** 1903-03-07

**Authors:** 


### PROGRESS IN NEUROLOGY.

(*Concluded from page* 375.)

**Pathology of Nerve Degeneration.**—Mott,[6] in discussing this subject, says that the Marchi reaction depends on the splitting up of the complex phosphoretted fat lecithin into simpler bodies, such as choline, glycero-phosphoric acid, and stearic or oleic acid. The choline and glycero-phosphoric acid escape into the blood, whilst the non-phosphoretted fat is left behind and is blackened by the action of the osmic acid. The choline can be detected in the blood; 5 c.cm. of blood can be taken direct from a vein by venesection or preferably with a sterile hypodermic syringe. It should be mixed with six or eight times its volume of absolute alcohol, allowed to stand for some time, stirred and filtered. The alcoholic filtrate is evaporated to dryness at 40° C., and the residue taken up with absolute alcohol. This is repeated twice. One drop of platinum chloride solution (10 per cent. in alcohol) is added. The precipitate which forms is washed with absolute alcohol, and then dissolved in 15 per cent. alcohol, filtered and evaporated to dryness. Microscopical examination shows the presence of yellow octahedral crystals of the double salt of platinum and choline. This test shows the presence of choline in the blood not only where experimental degeneration of nervous tissues, *e.g.* of the sciatic nerves, is produced in an animal, but also in degenerative diseases of the nervous system in man, for instance, beriberi, alcoholic neuritis, disseminated sclerosis, and general paralysis. It may be of considerable utility in deciding whether a nervous disease is functional or organic in nature. Choline is a cardiac depressant, and its presence in the blood may, in cases of degenerative nervous diseases, assist in causing a fatal termination.

**African Lethargy.**—Warrington examined the central nervous system from a case of African lethargy. The patient, a girl of about 19 years of age had been ill five months, she gradually became more and more sleepy, then weaker, and died without presenting any other symptoms; there were no abnormal physical signs present in any organ of the body. The dura mater was normal, whilst the pia-arachnoid was slightly thickened and infiltrated with round cells. The perivascular lymphatics of the central nervous system were distended with lymphocytes. The nerve-cells were normal, and only in the spinal cord was there any trace of degenerated nerve-fibres. The condition was thus, anatomically, a subacute leptomeningitis. What the poison is, however, which causes this condition is as yet unknown.

**Kernig's Sign** is found in nearly all cases of meningitis, and if present has been thought pathognomic of that affection. It may be absent, however, in cases proved to be meningeal by post-mortem examination.[8] Cases have been reported in which it was present though no meningitis was found. Thus Widal and Merklen reported its presence where the lesion was a clot pressing on the anterior surface of the pons and medulla, and Thyne where the lesion was a hæmorrhage into the left lateral lobe of the cerebellum and an effusion of blood over the right occipital lobe and into the fourth ventricle. This list has been further extended by Sailor,[9] who found it present unilaterally in two cases of focal encephalitis. The explanation of the phenomenon is doubtful. Some have supposed it to be a sign of irritation of the spinal meninges, while Chaufford thinks it is merely an exaggeration of a normal phenomenon due to a hypertonicity of the muscles. Sailor is of opinion that it is a symptom of a partial lesion of the pyramidal tract. It is clear that it is not a lesion of the meninges but of the subjacent nervous substance which causes Kernig's sign, and such a lesion may irritate though not destroying the pyramidal tracts.

**Landry's Paralysis.**—As the result of a study of cases of Landry's paralysis, Walton[10] comes to the following conclusions. It is an infection or toxæmia produced by various poisons in which the bulb, spinal cord, and peripheral nerves, and especially the lower motor neuron are attacked, and in which parenchymatous degeneration, hæmorrhagic and inflammatory exudutes of various kinds and degree, are usually found post-mortem in this portion of the nervous system. But in a few cases no post-mortem changes are discoverable, and in still others changes which are inadequate to explain the symptoms. The spleen and lymph glands are usually enlarged. Bacteria of various kinds have been found, but in the great majority of cases a careful examination has failed to discover any micro-organisms. The first symptom of the disease is usually paresthesia, sometimes accompanied by pain in the members which are subsequently paralysed, and this may exist from a few hours to several days as a premonitory symptom. Widespread flaccid paralysis rapidly develops, involving legs, trunk, and arms, and in fatal cases the muscles innervated from the bulb. The paralysis is usually of an ascending type involving the legs first, but it is occasionally of a descending type or may involve all extremities simultaneously. In the majority of cases the sphincters are intact. The knee-jerks are absent. As a rule, atrophy, fibrillary twitchings, and electrical changes in the muscles are absent. Pain and tenderness to pressure may be prominent symptoms, but as a rule are not very marked. Usually the temperature is normal or but slightly raised. Consciousness is almost invariably intact. Cases which terminate fatally run a course of from a few days to one or two weeks, and almost always exhibit bulbar symptoms. In cases which do not terminate fatally the course may extend over many weeks or even months, and recovery may be incomplete. The prognosis is always grave, though some recover. Very little can be done for the patient, treatment should be directed to the support and stimulation of the vitality and the production of elimination. To this end hypodermic injections of strychnia, and the use of whisky, digitalis, and mild diuretics and cathartics are to be recommended.

**Exophthalmic Goitre.**—Statistics of 120 cases of this disease have been given by Murray;[11] 110 were women and 10 were men. The onset was nearly always insidious, and generally between the ages of 15 and 35. Recent events producing powerful emotions had acted as exciting causes in some cases. The first symptom most commonly noted was enlargement of the thyroid. The frequency of the pulse was distinctly increased in all the cases, and in half of the number it was between 120 and 150 per minute. Exophthalmos was absent in one-quarter of the cases. Nervous tremour was a very constant sign, being present in 111 cases. Restlessness and various symptoms, such as suppressed excitement, hallucinations, and insomnia were also frequent; and in a considerable number paroxysmal exacerbations of these and other symptoms occurred. Diarrhœa or an increased frequency of defœcation was by no means uncommon. The prognosis of the disease was uncertain. Out of 40 cases seen from time to time, seven died, two remained stationary, and 31 progressed favourably. Rest in bed was advocated, and the Weir-Mitchell treatment was of great service. Faradic electricity gave relief, and in some cases resulted in improvement. Belladonna was disappointing, whilst extracts of thymus and suprarenal glands had both been of service. In discussing this paper Hale White said that a considerable proportion of his patients had suffered from polyuria, which was often an early symptom. In fatal cases Peyer's patches were frequently found enlarged. Mackenzie said that about 6 per cent. of cases had a history of rheumatism. Tremors and psychical symptoms were most important and frequent phenomena. Suspicion was the leading feature of the insanity which was so often present in the disease. It was very easy to produce glycosuria by administration of glucose.

**Typhoid Psychosis.**—Some interesting cases and a *resumé* of the subject have been given by Farrar,[12] who says that typhoid fever attacking a sane person may leave him free from psychic symptoms or give him all grades of mental disease. The severity of the symptoms does not necessarily stand in relation to the height of the fever or the profoundness of the infection. Persons of psychopathic heredity are more prone to alienation, epecially initial delirium, than those not thus burdened. Initial delirium is the rarest form, exhibits the most rapid course and the worst prognosis, over 50 per cent. ending fatally. It often causes error in diagnosis. Any case of mental derangement with fever justifies the suspicion of typhoid. This initial delirium is essentially the expression of severe intoxication. Febrile psychosis are of the greatest frequency and afford the best outlook; over 25 per cent., however, persist for varying times into an after convalescence; they are especially due to elevation of temperature and its consequences. Asthenic psychoses, occurring during convalescence, present long weary courses and a doubtful outlook, with evidences of serious cerebral changes. They develop upon a basis of exhaustion, anæmia, and malnutrition. The typhoid psychosis is not distinctive either in its clinical or its anatomical picture. The elements of intoxication, infection, temperature, and exhaustion, of whatever origin, may produce similar or indistinguishable appearances. The determining factor of susceptibility to mental disorder may be expressed as the mental reaction coefficient of the individual together with its physical basis.

**Gastric Vertigo** is described by Fischer[13] as presenting the character that everything whirls round the patient like a revolving wheel. There was no loss of consciousness and no misconception of the hallucinations. Nausea usually follows the attack and is associated with pain in the stomach radiating to the back. Gastric vertigo is not associated with any one well-defined pathological condition of the stomach, or with a special abnormality of the gastric secretion. In regard to the diagnosis, it has to be remembered that vomiting is a common accompaniment of vertigo, whatever its origin, so that the mere presence of nausea and vomiting is not sufficient to establish the diagnosis of gastric vertigo.

[6] Brit. Med. Jour., Sept. 27. [7] Ibid. [8] Amer. Jour. Med. Sci., May. [9] Ibid. [10] Jour. of Nerv. and Ment. Dis., Oct. [11] Brit. Med. Jour., Nov. 1. [12] Amer. Jour. of Ins., July. [13] Med. Rec., June 7.

